# High-Throughput Screening Assay for Laccase Engineering toward Lignosulfonate Valorization

**DOI:** 10.3390/ijms18081793

**Published:** 2017-08-18

**Authors:** David Rodríguez-Escribano, Felipe de Salas, Isabel Pardo, Susana Camarero

**Affiliations:** 1 Centro de Investigaciones Biológicas, CSIC, Ramiro de Maeztu 9, 28040 Madrid, Spain; davidre04@gmail.com (D.R.-E.); fsalas@cib.csic.es (F.d.S.); Isabel.Pardo@nrel.gov (I.P.); 2 National Renewable Energy Laboratory, 15013 Denver West Parkway, Golden, CO 80401, USA

**Keywords:** laccase, lignosulfonate, high-throughput screening, phenolic content, enzyme directed evolution

## Abstract

Lignin valorization is a pending issue for the integrated conversion of lignocellulose in consumer goods. Lignosulfonates (LS) are the main technical lignins commercialized today. However, their molecular weight should be enlarged to meet application requirements as additives or dispersing agents. Oxidation of lignosulfonates with fungal oxidoreductases, such as laccases, can increase the molecular weight of lignosulfonates by the cross-linking of lignin phenols. To advance in this direction, we describe here the development of a high-throughput screening (HTS) assay for the directed evolution of laccases, with lignosulfonate as substrate and the Folin–Ciocalteau reagent (FCR), to detect the decrease in phenolic content produced upon polymerization of lignosulfonate by the enzyme. Once the reaction conditions were adjusted to the 96-well-plate format, the enzyme for validating the assay was selected from a battery of high-redox-potential laccase variants functionally expressed in *S. cerevisiae* (the preferred host for the directed evolution of fungal oxidoreductases). The colorimetric response (absorbance at 760 nm) correlated with laccase activity secreted by the yeast. The HTS assay was reproducible (coefficient of variation (CV) = 15%) and sensitive enough to detect subtle differences in activity among yeast clones expressing a laccase mutant library obtained by error-prone PCR (epPCR). The method is therefore feasible for screening thousands of clones during the precise engineering of laccases toward valorization of lignosulfonates.

## 1. Introduction

**Figure 1:**
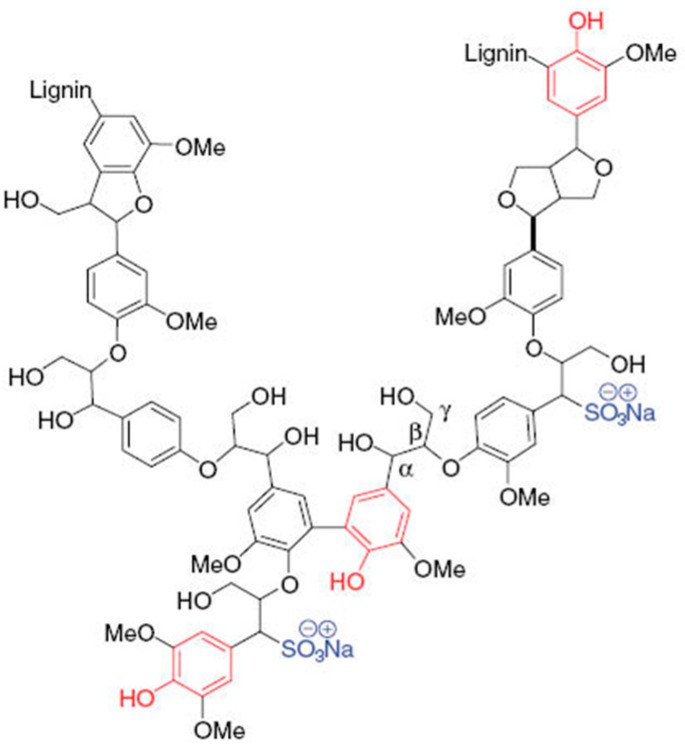
General chemical structure of lignosulfonate. Phenolic groups are depicted in red and sulfonate groups in blue.

Conversion of waste products of the pulping processes, such as lignin, into valuable products is being considered as one of the main objectives to increase the economic viability of lignocellulosic biorefineries. Huge quantities of “technical lignins” are produced in cellulose-pulping operations for the production of paper. Currently, sulfite and kraft processes are the dominating pulping technologies to extract the cellulosic fibers from wood and annual plants. The sulfite process isolates cellulose by sulfonating the lignin with sulfourous acid and/or a sulfite salt, which solubilizes and extracts the lignin. The resulting technical lignins are denoted lignosulfonates (LS), which are water-soluble polyphenolic macromolecules with sulfonate groups on the aliphatic side chain (Figure 1). Due to their unique properties, LS have an established market as additives in concrete, but are also used as road or animal feed binders or as dispersants in dyes, pesticides and other applications [1]. Currently, LS account for 90% of the total market of commercial lignin. About 1 million tons of LS are produced annually, with Borregaard-LignoTech (Sarpsborg, Norway) and Tembec (Montréal, QC, Canada) the dominant producers [2].

Due to the intrinsic heterogeneity of the lignin polymer and its degradation during the pulping process, commercial LS show wide molecular weight profiles, with molecular mass (*M_r_*) values roughly between 5000–20,000 Da (although much lower and higher values are also found). LS can be further modified before use to alter the dispersing, binding or complexing properties required for particular applications. Small *M_r_* lignosulfonates are preferred for phenolic resins synthesis [3], whereas large *M_r_* lignosulfonates are beneficial for use as dispersing agents, for example, in dye solutions exerting better dispersion and heat stability [4]. In line with this demand, oxidation of LS with ligninolytic oxidoreductases can promote cross-linking reactions that uniform and enlarge the size of lignin molecules, thus improving the usage of these technical lignins as dispersants. Large *M_r_* LS have been obtained through polymerization with laccases and peroxidases, with the advantage for laccases over peroxidases of using oxygen from the air as the only requirement [5,6,7,8,9].

Laccases (EC 1.10.3.2) oxidize directly the phenolic components of lignin, and indirectly—in the presence of a proper redox mediator—the phenolic and heterogeneous nonphenolic (especially methoxybenzene) components. As a result, radicals are generated in lignin, which can lead to aliphatic or aromatic C–C bond cleavage and depolymerization [10,11]. However, spontaneous repolymerization of the quinonoid radical intermediates typically counteracts the depolymerization effect. In fact, polymerization prevails in all laccase treatments of lignosulfonates described so far. The oxidation of free phenolic end-groups into phenoxyl radicals which, in turn, undergo radical-coupling reactions, produces a notable decrease of phenolic content and an increase of lignin *M*_r_, simultaneously reducing the heterogeneity of sizes of polymer molecules [5,6,7].

With this information in mind, and with the aim of improving LS polymerization by laccase (in the absence of costly redox mediators), we describe here the development of a high-throughput screening assay for the precise engineering of fungal laccases towards LS, based on phenolic content measurement.

## 2. Results

### 2.1. Treatment of Lignosulfonates with Laccase: Decrease of Phenolic Content

**Figure 2:**
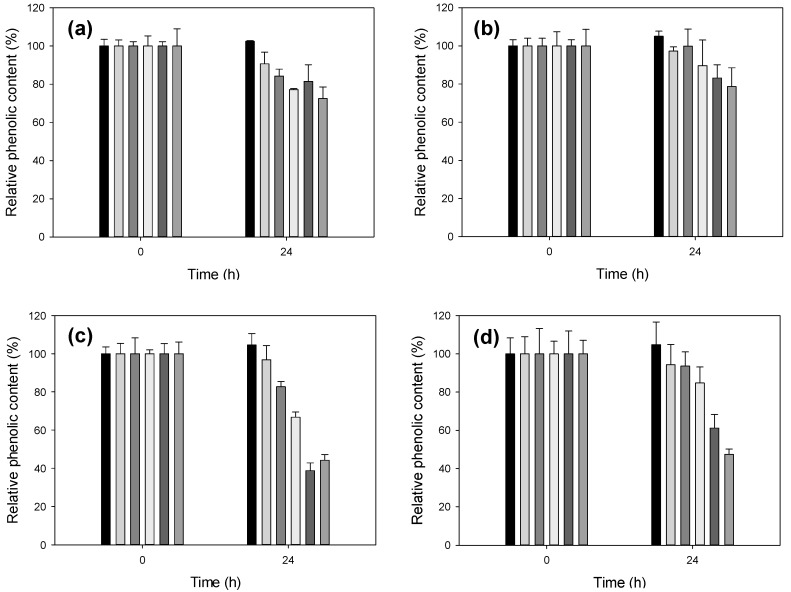
Variation in the relative phenolic content of lignosulfonates DP399 5 g/L (**a**) or 10 g/L (**b**), and DP401 5 g/L (**c**) or 10 g/L (**d**), after 24 h of reaction with different amounts of LAC7. From left to right: 0, 5, 10, 20, 50 and 100 mU/mL of laccase activity. Error bars indicate standard deviation for three replicates.

Lignosulfonates (LS, 5–10 g/L) from softwood (DP399) and hardwood (DP401) were oxidized with different activity units of a fungal high-redox-potential laccase (LAC7) previously obtained [12]. Changes in lignin phenolic content over time were monitored by using the Folin-Ciocalteau reagent (FCR), which reacts with the free hydroxyl groups, giving a colorimetric response with maximum absorbance at 760 nm [5]. The reaction mixture/FCR ratio was optimized to attain the most suitable (i.e., measurable) colorimetric response. Optimal absorbance at 760 nm was reached by using 10 µL of the reaction sample and 15 µL of FCR (with Na_2_CO_3_). In general, the higher the laccase activity used, the lower the phenolic content of the treated lignosulfonate. The decrement in phenolic content was more evident at 24 h of treatment (no significant differences were obtained with shorter incubation times, whereas longer ones did not enhance the colorimetric response). Enzymatic effect was more marked when using 5 g/L than 10 g/L of LS, and when using DP401 (a 40–60% decrease of initial phenolic content with 50 U/L enzyme) than DP399 (a 20–30% reduction of phenolic content with the highest laccase activity assayed) (Figure 2).

### 2.2. Comparison of Different Recombinant Laccases

**Figure 3:**
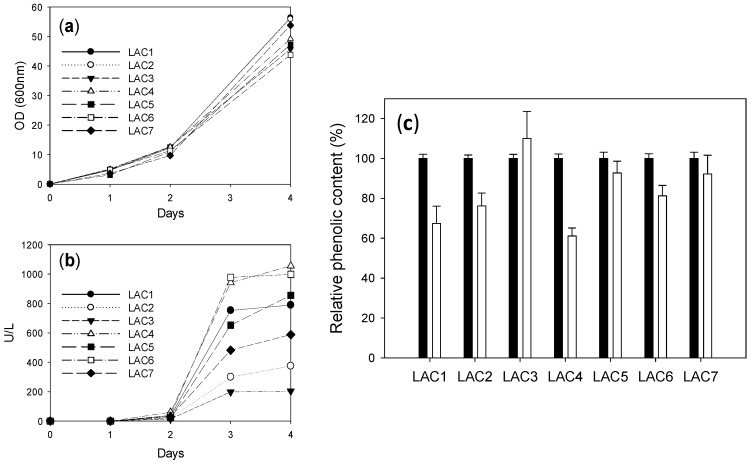
Optical density (OD) at 600 nm (**a**) and laccase activity (with 3 mM ABTS pH 5) (**b**) in *S. cerevisiae* cultures producing selected laccase variants (flasks). Relative phenolic content in DP401 lignosulfonate before (black bars) and after 24 h treatment with concentrated crudes of these laccases (20 mU/mL) (white bars). Error bars indicate standard deviation for three replicates (**c**).

Once the conditions for the enzymatic treatment of LS were set, different fungal laccase variants previously engineered in the lab (LAC1 to LAC7) [12,13,14] were produced in *S. cerevisiae*, and their capabilities to oxidize DP401 were compared. Optical densities (OD 600) were quite similar for all *S. cerevisiae* clones expressing the diverse laccases (Figure 3a), whereas important differences were observed among laccase activities detected in the liquid cultures. The highest laccase activities detected (3–4 days) corresponded to LAC4 and LAC6 (Figure 3b).

Then, crude enzymes were concentrated and compared for LS oxidation using 20 mU/mL of each one (activities measured with 2,2′-azino-bis(3-ethylbenzothiazoline-6-sulphonic acid), ABTS) (Figure 3c). LAC4 and LAC1 were the most efficient laccases oxidizing (polymerizing) LS as determined by the decrease in phenolic content. Both enzymes were selected for the next assay.

### 2.3. Polymerization of Lignosulfonate by Laccase with or without Redox Mediators

**Figure 4:**
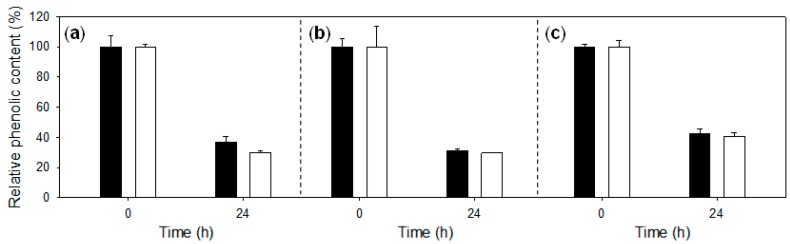
Variations in the relative phenolic content of lignosulfonate DP401 after treatment with LAC4 (black) and LAC1 (white) (25 mU/mL) for 24 h, without a mediator (**a**) or in the presence of violuric acid (**b**) or 4-hydroxybenzoic acid (**c**). Error bars indicate standard deviation for three replicas.

The effect of two redox mediator compounds, 4-hydroxybenzoic acid (as a phenolic mediator) and violuric acid (as a synthetic mediator), on the enzymatic polymerization of LS DP401 by LAC4 and LAC1 was tested for 24 h (Figure 4). A strong decrease in LS phenolic content (measured by the FCR method) was observed with both enzymes. The final phenolic content obtained with LAC4 and LAC1 was quite similar (differences below 5%). None of the two mediators enhanced the results obtained with the two laccases alone. Therefore, we proceeded with the initial hypothesis of developing a HTS assay to improve LS polymerization by laccase in the absence of costly redox mediators.

### 2.4. Comparison of Laccase Variants Produced in S. cerevisiae Microcultures for LS Polymerization

**Figure 5:**
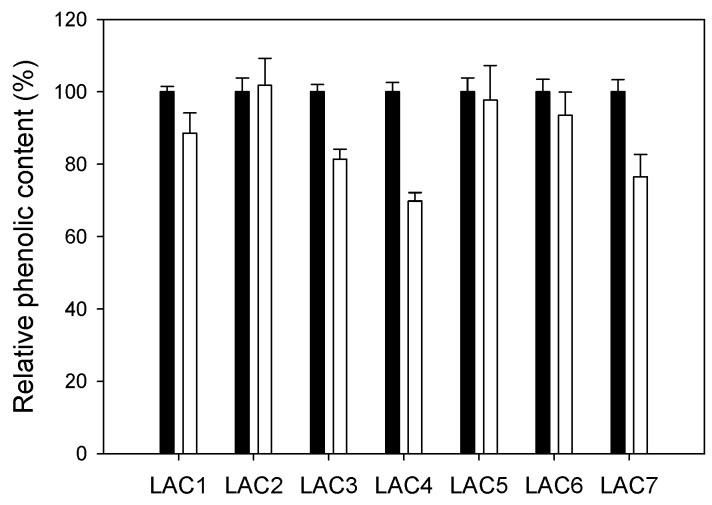
Relative phenolic content in DP401 lignosulfonate before (black bars) and after 24 h-treatment with 20 µL supernatants from *S. cerevisiae* microcultures secreting the different engineered laccase variants (white bars). Error bars indicate standard deviation for three replicates.

To verify if these results could be transferred to a HTS format for exploration of laccase activity in mutant libraries, *S. cerevisiae* clones expressing the different laccase variants were cultured in microplates. Supernatants (20 μL) from these microcultures were mixed with 180 μL LS DP401 for 24 h, and the reaction was thereafter revealed with FCR. Differences in laccase activities secreted by the different yeast clones were evidenced by dissimilar decreases in LS phenolic content (Figure 5). Although LAC4 and LAC1 had shown similar activities with LS in the assay with crude enzymes, the higher production of LAC4 by the yeast (illustrated in Figure 3b) led, in this case, to a superior total activity in the supernatant and, consequently, produced the highest reduction of LS phenolic content. We therefore selected LAC4 to validate the HTS colorimetric assay.

### 2.5. LS-FCR High-Throughput Screening Assay

**Figure 6:**
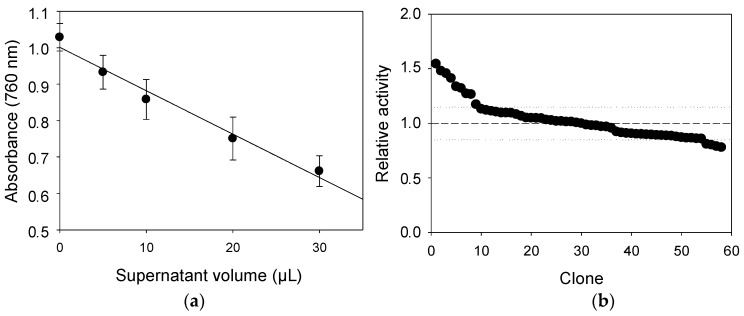
Linearity of the high throughput screening (HTS) colorimetric assay with increasing volumes of supernatant from *S. cerevisiae* microcultures secreting LAC4 (**a**); reproducibility of the assay shown by the relative activities of 60 replicas of *S. cerevisiae* clones secreting LAC4 (dotted lines indicate CV) (**b**).

First, linearity of the high-throughput screening (HTS) colorimetric assay was evaluated with different supernatant volumes of *S. cerevisiae* microcultures secreting LAC4 (using 5 g/L LS DP401 in 24 h reactions). The colorimetric response (absorbance at 760 nm) developed with FCR was linear in the range of 0–30 μL supernatant in the reaction (Figure 6a). Thus, 20 μL was fixed as a volume adequate to give an accurate colorimetric response and to be sampled with the liquid-handling robot. Uncertainty of the HTS assay was estimated by replicating and culturing *S. cerevisiae* cells expressing LAC4 in a 96-well plate (60 colonies). The variability of laccase activities measured with LS-FCR in each well (coefficient of variation (CV) = 15%) proved the reproducibility of the assay (Figure 6b) [15].

**Figure 7:**
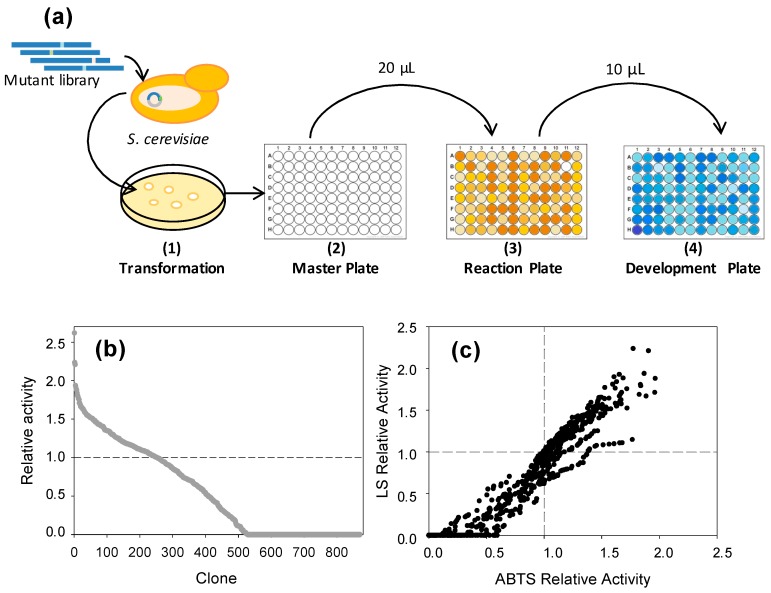
Scheme of the HTS protocol (**a**) including generation of the laccase mutant library and transformation in yeast (1), library expression in *S. cerevisiae* microcultures (2), transfer of supernatants to replica plates and reaction with LS (3), and color development by reaction of free phenolic groups with FCR (4). Landscapes for the activities of the clones of the mutant library screened with LS-FCR (**b**) or ABTS (**c**). Activity of parental LAC4 is depicted as dashed lines.

Next, we aimed to demonstrate the feasibility and sensitivity of the HTS colorimetric assay to screen mutant laccase libraries in directed evolution campaigns. For this, a mutagenic library was created by error-prone PCR (epPCR) of LAC4 and screened using the LS-FCR method. The optical (but not easily measurable) response of the reaction plates, due to darkening of LS by oxidation with laccase, correlated with a decrease of the blue color in FCR plates due to reduction of phenolic content (Figure 7a). Landscape from the screening with LS showed significant differences among the activities of the clones, some of them with over two-fold total activity improvements with respect to the activity of the parental (Figure 7b). Laccase activities in the library were also measured with ABTS as a reference method. In general, a correlation between the activities of the clones with LS and ABTS was observed, although some of the most active clones showed trends towards one or the other substrate (Figure 7c). The accuracy of the HTS assay to detect improved mutants respecting the parent type was therefore demonstrated.

## 3. Discussion

Enzymatic polymerization of lignosulfonates with laccases is based on the ability of these enzymes to oxidize the phenolic groups of lignin and form phenoxyl radicals. On a molecular level, different studies have demonstrated how the increase in the molecular weight of LS can be enhanced by the introduction of radicals on the phenolic end groups of LS and their subsequent coupling, leading to cross-linking of different LS molecules imitating the natural lignin biosynthesis. Condensation reactions, between the different resonance forms of the radicals generated on the polymer chain by enzymatic oxidation of phenolic units, result in the formation of new ether and C–C (aryl–aryl or aryl–alkyl) linkages, thus causing LS polymerization and the decrease in lignin phenolic content. In contrast with other oxidoreductases, such as peroxidases, laccases are able to oxidize phenolic end groups in lignin, but in principle, they leave the non-phenolic end groups untouched (which constitute the main fraction in lignin polymer). Only the presence of redox mediator compounds enables the oxidation of the most recalcitrant lignin moiety (non-phenolic lignin units) by laccase [16].

For a lignin-degradation purpose (which is of high relevance from an economic point of view), the sole oxidation of lignin polymer with laccases in the absence of redox mediators may not be of advantage. On the contrary, the oxidation of lignosulfonates by laccase alone represents a benefit if lignin polymerization is desired. LS polymerization by laccase is caused by the formation of radicals which couple into phenyl ether–carbon or carbon–carbon bonds. These bonds generate intramolecular linkages within the LS molecule, and intermolecular linkages with other LS molecules. The resulting lignosulfonates are more homogeneous in size, with larger *M_r_*, and consequently, expanded commercial usage [17]. The increment in *M_r_* inversely correlates with the phenolic content of the sulfonated lignin after the enzymatic treatment [18]. In the present study, the higher decrease of phenolic content in DP401 over DP399 after laccase treatment may be related to the micelles-structure formation of LS in water. Enzyme access is restricted to the phenolic end groups exposed on the LS micelle surface (and not to the buried ones) due to steric constraints [5]. Thus, the laccase effect is much more notable in lower initial *M_r_* LS such as DP401, resulting in a more prominent decrease in phenolic content than in LS DP399. Along these lines, laccase treatment of different technical lignins (including kraft lignins, lignosulfonates or steam-exploded lignin solutions) has demonstrated better reactivity of lignins with components of lower molecular mass and higher phenolic content [19].

LAC1 and LAC4 showed the highest activities on LS DP401 as compared with other laccase variants developed in the laboratory [12,13,14]. The phenolic content of LS was reduced by more than 40% after treatment with both laccases, whereas the phenolic content diminished less than 30% with the rest of the laccases tested. Although no significant differences were found between LAC1 and LAC4 activities on LS (Figure 3c), significant differences were observed in laccase production by *S. cerevisiae* (with LAC4 being significantly better-produced than LAC1). Therefore, the total laccase activity detected in the supernatants from yeast microcultures (the format in which the HTS of mutant libraries is performed) was significantly higher for the clone expressing LAC4. It is important to highlight that, as the enzyme is secreted by the yeast in its active form, in directed evolution studies total laccase activity comprises the sum of catalytic activity and enzyme expression (secretion). Thus, it is convenient to select a starting point with sufficient total activity so as to facilitate the proper validation of the method assay, including the screening of a laccase mutant library.

On the other hand, no beneficial effects with any of the enzymes tested were observed when LS were oxidized by laccase in the presence of redox mediators such as violuric acid or 4-hydroxibenzoic acid (HBA). In fact, LS DP401 phenolic content after reaction with laccase-HBA seemed to be faintly higher than after enzymatic reaction without a mediator, suggesting less polymerization. Size-exclusion chromatography (SEC) analyses will be carried out to corroborate the polymerization degree obtained with laccase (with or without mediators). However, Fourier-transform infrared spectroscopy (FTIR) analysis revealed notable changes of lignin bands after LS oxidation by laccase, suggesting polymerization and the formation of bigger aggregates [8] (see Figure S1). Different studies have evidenced LS polymerization by laccase in the presence of synthetic or natural mediators [7,20]. Nevertheless, we demonstrated here that the presence of redox mediators does not promote the action of laccase on LS, as has been lately described [17], suggesting that mediators might indeed counteract polymerization by promoting degradation reactions. Lignosulfonate *M*_r_ has been considerably augmented by direct oxidation with fungal laccases (without mediators) [21], particularly when high-redox-potential laccases are applied [5]. The enhancement of lignosulfonate-dispersing properties by direct oxidation with high-redox-potential laccases, in the absence of expensive and possibly toxic redox mediator compounds, is beneficial from an industrial point of view.

## 4. Materials and Methods

Determination of phenolic content by the Folin–Ciocalteau reagent. Lignosulfonates (DP401—hardwood, *Eucalyptus grandis*, with *M_r_* around 6000 Da—and DP399—softwood, *Picea abies*, with *M*_r_ around 46,000 Da) were supplied by Borregaard-LignoTech (Sarpsborg, Norway). Folin–Ciocalteau reagent (FCR), obtained from Panreac (Barcelona, Spain), was used to determine phenolic content in lignosulfonates by adapting the protocol from Areskogh et al. [5]. For that, 10 μL samples of lignosulfonates were mixed in 96-well plates with 15 μL 0.135–0.165 M FCR in 200 μL final volume for 5 min. Then, 50 μL of Na_2_CO_3_ (20 % *w*/*v*) was added and plates were incubated with low agitation until the development of a blue color (1 h). Afterwards, absorbance at 760 nm was measured in a SPECTRAMAX Plus 384 plate reader (Molecular Devices, Sunnyvale, CA, USA).

Production of laccase variants in yeast (flasks). pJRoC30 plasmids carrying the selected laccase genes (LAC1–LAC7, including evolved α pre-proleaders) were obtained in previous works from a high-redox-potential laccase from basidiomycete PM1 (*Coriolopsis* sp.) [12,13,14,22]. Protease-deficient BJ5465 *S. cerevisiae* clones transformed with the different laccase genes were grown in 100 mL flasks with 30 mL expression medium (containing Cu_2_SO_4_ and ethanol) [22], in duplicate, at 30 °C and 220 rpm for 4 days. Laccase activity secreted in the liquid cultures was monitored over time by measuring the oxidation of 3 mM ABTS (2,2′-azino-bis(3-ethylbenzothiazoline-6-sulfonic acid)) in 100 mM citrate–phosphate (CP) buffer pH 5. After 4 d of incubation, cultures were centrifuged at 5000*× g* for 10 min and supernatants were filtered through 0.8 and 0.45 μm-pore-size membranes. Subsequently, filtered supernatants were concentrated 10-fold in Amicon Ultra 10,000 MWCO centrifugal units (Millipore, Billerica, MA, USA).

Treatment of lignosulfonates with laccase: selection of reaction conditions. Concentrations of 5 and 10 g/L of lignosulfonates DP401 and DP399 dissolved in 125 mM CP buffer pH 5 were treated with different activity units (0–100 U/L) of crude laccase (measured with 3 mM ABTS, pH 5), in 200 μL reaction volume. After 0, 6, 24 and 48 h of enzymatic treatment at 28 °C and 180 rpm (80% humidity), 10 μL samples were drawn and the phenolic content was determined with the FCR method as described above. Oxidation of LS with laccase with or without redox mediators was carried out with LS DP401 (5 g/L) in 125 mM citrate–phosphate buffer, pH 5 (200 μL final volume) with 20 or 25 mU/mL of crude laccase. Violuric acid and 4-hydroxybenzoic acid (both from Sigma-Aldrich, Saint Louis, MO, USA) were added in 1.7 mM final concentration as mediators. Reactions were carried out for 24 h before determination of phenolic content.

*S. cerevisiae* microcultures expressing different laccases. Conditions for transformation and growth of the aforementioned *S. cerevisiae* clones and for laccase expression in liquid microcultures have also been defined [22]. For the evaluation of laccase activity, plates were centrifuged and 20 μL supernatant from each well was transferred into replica plates using a liquid-handling robot (Quadra96, Tomtec, Hamden, CT, USA). Activities were measured with LS DP401, as mentioned in the previous section.

Construction of the laccase mutant library. The mutagenic library was obtained by epPCR of the LAC4 gene using GeneMorph II Random Mutagenesis Kit (Agilent Technologies, Santa Clara, CA, USA) with the conditions described for medium mutational frequency (1–3 mutations/kb). LAC4 was amplified from the pJRoc30 plasmid using primers FORW EXT sense (5′-CTGGGGTAATTAATCAGCGAAGC-3′) and REV EXT antisense (5′-CCAAAACCTTCTCAAGCAAGG-3′), which generate overhangs which anneal (40–66 bp regions) with the ends of the linearized vector. An amount of 400 ng of purified PCR product was cotransformed with 100 ng of the linearized vector into competent yeast cells for in vivo cloning and recombination. General considerations regarding PCR conditions and purification of amplified product have been previously described [12].

Validation of the HTS colorimetric assay with LS-FCR. Linearity of the assay was tested in triplicate with different supernatant volumes (5–40 μL) of a microculture of *S. cerevisiae* expressing LAC4. Coefficient of variation (CV) was determined by using 20 μL supernatants of a plate in which every well was inoculated with yeast cells also expressing LAC4. Conditions for the reaction with LS DP401 and FCR were the same as described above.

High-throughput screening of a laccase mutant library. Around 900 clones of the LAC4 mutant library were analyzed using 20 μL supernatants and LS DP401 and FCR as aforementioned. Relative activities were calculated by the decrease of absorbance at 760 nm with respect to initial absorbance values, and normalized with respect to the activity of the parent type. The mutant library was also screened with 180 μL of 3 mM ABTS in acetate buffer pH 5. In order to elude false positives, two consecutive rescreenings were carried out as previously described [22].

## 5. Conclusions

The intrinsic ability of laccase to oxidize lignin phenols and catalyze cross-linking reactions was used here to develop a HTS assay for the directed evolution of fungal laccases toward the polymerization of lignosulfonates. The HTS assay, based on colorimetric detection of phenolic content by FCR, is rapid, reproducible, feasible and sensitive enough to discriminate among modified activities from laccase mutant libraries. Enhancement of enzymatic polymerization of LS without costly mediators is relevant for expanding both the commercial usage of these technical lignins and for laccase industrial application. Moreover, the method would also be applicable for the accurate detection of phenol release during the enzymatic depolymerization of lignin, a pending biotechnological goal for lignin valorization.

## Supplementary Materials

Supplementary materials can be found at www.mdpi.com/1422-0067/18/8/1793/s1.

## Abbreviations

ABTS	2,2′-Azino-bis(3-ethylbenzothiazoline-6-sulfonic acid)
CV	Coefficient of variation
DP399	Lignosulfonate from softwood (*Piceaabies*)
DP401	Lignosulfonate from hardwood (*Eucalyptus grandis*)
epPCR	Error-prone PCR
FCR	Folin–Ciocalteau reagent
HRPL	High-redox-potential laccase
HTS	High-throughput screening
LAC	Laccase
LS	Lignosulfonates
*M*_r_	Molecular weight
