# Supplementary material for: High-Throughput Screening Assay for Laccase Engineering toward Lignosulfonate Valorization
Source: Int J Mol Sci. 2017 Aug 18;18(8):1793. doi: 10.3390/ijms18081793 (PMC5578181; doi:10.3390/ijms18081793)
Supplement: Supplementary file 1 [file ijms-18-01793-s001.pdf]

Supplementary figure

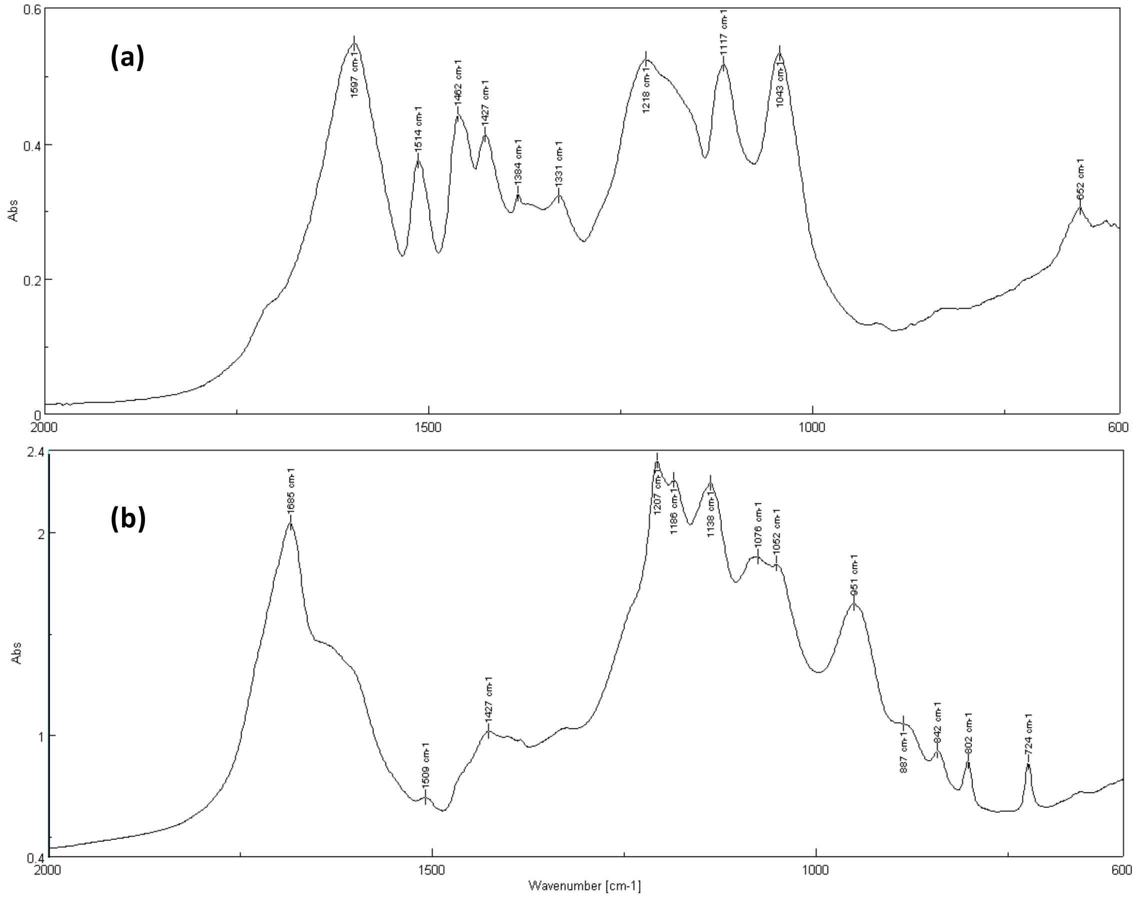

Figure S1. FTIR spectra for DP401 lignosulfonate before (**a**) and after treatment with laccase (**b**), showing significant changes after lignin oxidation by the enzyme such as: i) decrease of the intensity of the band around 1220 cm^{-1} corresponding to S=O groups and of the distinct band at 652 cm^{-1} assigned to stretching vibration of sulfonic groups; ii) replacement of the lignin's characteristic bands between 1600- 1425 cm^{-1} (assigned to C=C skeletal vibrations, aromatic ring vibrations and C-H deformation), by two large peaks at this region, the new peak at 1685 cm^{-1} corresponds to unconjugated carbonyl-carboxyl stretching; iii) changes in 1331 cm^{-1} band reported for syringyl rings (predominant in hardwood lignin) ; iv) increases in intensity of bands appeared at 1138 and 802 cm^{-1}, associated to CH deformations (in plane and out of the plane, respectively); and v) new peak detected at 950 cm^{-1} related to the C–C stretch of aliphatic chains obtained after oxidation by laccase.
